# Exploring the boundaries: gene and protein identification in biomedical text

**DOI:** 10.1186/1471-2105-6-S1-S5

**Published:** 2005-05-24

**Authors:** Jenny Finkel, Shipra Dingare, Christopher D Manning, Malvina Nissim, Beatrice Alex, Claire Grover

**Affiliations:** 1 Department of Computer Science, Stanford University, Stanford CA 94305-9040, USA; 2 Institute for Communicating and Collaborative Systems, University of Edinburgh, United Kingdom

## Abstract

**Background:**

Good automatic information extraction tools offer hope for automatic processing of the exploding biomedical literature, and successful named entity recognition is a key component for such tools.

**Methods:**

We present a maximum-entropy based system incorporating a diverse set of features for identifying gene and protein names in biomedical abstracts.

**Results:**

This system was entered in the BioCreative comparative evaluation and achieved a precision of 0.83 and recall of 0.84 in the "open" evaluation and a precision of 0.78 and recall of 0.85 in the "closed" evaluation.

**Conclusion:**

Central contributions are rich use of features derived from the training data at multiple levels of granularity, a focus on correctly identifying entity boundaries, and the innovative use of several external knowledge sources including full MEDLINE abstracts and web searches.

## Background

The explosion of information in the biomedical domain and particularly in genetics has highlighted the need for automated text information extraction techniques. MEDLINE, the primary research database serving the biomedical community, currently contains over 14 million abstracts, with 60,000 new abstracts appearing each month. There is also an impressive number of molecular biological databases covering an array of information on genes, proteins, nucleotide and amino acid sequences, both generally (GenBank, Swiss-Prot) and for particular species (FlyBase, Mouse Genome Informatics, WormBase, Saccharomyces Genome Database), each containing entries numbering from the thousands to the millions and multiplying rapidly. All of these resources are largely curated by hand by expert annotators at enormous expense and the amount of information often prohibits updating previously annotated material to conform to changing annotation guidelines. This situation has naturally led to an interest in automated techniques for problems such as topic classification, word sense disambiguation, and tokenization in the biomedical domain (cf. MEDLINE's Indexing Initiative [1]).

In this paper we focus on the particular problem of Named Entity Recognition (NER) which requires the identification of names corresponding to shallow semantic categories. As posed by the BioCreative evaluation in Task 1A, this task required participants to identify a single entity "NEWGENE" corresponding roughly to gene and protein names in medical abstracts. NER is an important component for more complex information extraction tasks such as automatic extraction of protein-protein interaction information. We present a system based on a maximum-entropy sequence tagger which achieved state-of-the-art performance in the BioCreative comparative evaluation. Below, we first describe the system, then present its performance on the BioCreative Task 1A development and evaluation data along with an analysis of errors, and finally close with a more general discussion of the task and our conclusions.

## Implementation

Our entry was a machine learning system using a discriminatively trained sequence tagger. We devoted most of our efforts to finding useful features. The final system makes exhaustive use of clues within the sentence, as well as using various external resources, and pre- and post-processing. Below, we describe our system in greater detail. We outline the machine learning model, our preprocessing phase, and then we detail our full feature set, starting with the features used in the closed section of the BioCreative evaluation (where gazetteers were not allowed) and moving on to the features used in the open section (where all external resources were allowed). Then we give implementation details of our training procedure, and finally we describe tagging and a postprocessing phase aimed at improving boundary detection.

### Model

The model used was a conditional Markov model sequence tagger, implemented in Java and based on the tagger used in [2]. The system essentially uses a logistic regression model to put a probability distribution over the set of classes  = {NEWGENE, O} for each word. That is, for deciding the probability of class *c *at a certain word position, one employs a loglinear model that uses features *f*_*j *_of the input data  and previous classifications  to define the probability of the class as follows:



This calculation is then overlaid with a Viterbi-style dynamic programming algorithm [3] to find the best sequence of classifications. Such models are commonly referred to as maximum entropy models in the NLP literature [4,5] and are also known as maximum entropy Markov models or MEMMs [6]. Maximum entropy models have been used with much success in NER tasks and are known for their ability to incorporate a large number of overlapping features. The features used in our model are all binary indicator functions that pick out particular data contexts and pair them with each class. This restriction is not required by the model form, but gives the model a particularly simple semantics: in the model above one is simply summing the *λ*_*j *_weights for the features that "matched" (that is, have value 1) in a particular instance). For example, the matching features of the data context might be something like {prev. word = *murine*, curr. word = *CD4*, prev. class = O} – though in practice our model would typically have several dozen features matching at any position. As is common for NLP models using many features, we employ equal-scale quadratic regularization of the parameter weights to prevent parameters rarely present in the data having high weights, which leads to model overfitting. Modulo this penalization, the model parameters *λ*_*j *_are set to maximize the conditional likelihood of the class sequence on the training data.

### Preprocessing

During both training and testing we used the tokenization supplied by the task organizers. This tokenization was of quite poor quality. For instance, periods were always separated off as tokens, and so a text string like *[increased] by 1.7-fold*. was tokenized as . However, we kept with this tokenization for practical reasons: since evaluation was to be done with respect to this tokenization, introducing a pair of processes that mapped back and forth between this representation and another tokenization scheme seemed a potentially error-prone step that was unlikely to help final results.

We normalized names of months and days of the week to lowercase, and mapped the British spellings of a few common medical terms to their American versions. We looked up all tokens in the gazetteer and in the English dictionary CELEX and calculated the frequency of each token in the corpus. We then identified abbreviations and long forms using the method of [7]. We tagged the data for part-of-speech (POS) using the TnT POS tagger [8] trained on the GENIA corpus [9], which provides a gold standard for POS tags in biomedical text. The TnT POS tagger is an HMM-based tagger; perhaps due to greater robustness, we found that it outperformed the maximum entropy POS tagger that was available to us. Testing showed that a GENIA-trained POS tagger performed much better than one trained on *Wall Street Journal *text, due to the specialized nature of biomedical text. The task essentially required only picking out whether words were genes or not, but to allow recognition of adjacent but different named entities, the data made a NEWGENE versus NEWGENE1 distinction (in which the second of two adjacent but separate entities was labelled as NEWGENE1). We removed this distinction and mapped all entities to NEWGENE. Cases of adjacent named entities are sufficiently rare that it is hard to do well on them; we maximized performance by making the system unable to represent this situation.

### Features – closed section

The features described here were used in both the closed and open sections. The basic feature types were words, character substrings, word shapes, POS tags, abbreviations and the NE tags assigned to surrounding words. Character substrings refer to all substrings of the current word, up to a length of 6 characters. Thus the word "bio" would have features *_b, _bi, _bio, _bio_, bio_, io_, o_, bio, bi, io, b, i, o*. Word shapes refer to mappings of each word to a simplified representation that encodes attributes such as its length and whether it contains capitalization, numerals, greek letters, and so on. For example, "Varicella-zoster" would become *Xx-xxx*, "mRNA" would become *xXXX*, and "CPA1" would become *XXXd*. Beyond standard word and POS tag features, character substring and word shape features were central players in the system of [2]. A feature encoding whether each word was an abbreviation, a long form, or neither was assigned to each token. Lastly, a parentheses-matching feature that signalled when one parenthesis was classified differently from its pair was added in an effort to eliminate errors where the tagger classified matching parentheses differently. All of these basic feature types were then used singly or combined in various ways to create new features. Features matching a word were also used disjunctively on left and right contexts. We borrowed disjunctive word features from [10], and introduced abbreviation and parentheses matching features to model key problems in this textual domain. The resulting feature set is summarized in Table 1 and comprises all of the features used in the closed section.

### Features – open section

The features described here were used in the "open" entry and comprise various external resources including gazetteers, a web querying technique, the full abstracts corresponding to the sentences in training and test sets, the GENIA corpus, and the ABGene NE/POS tagger. The basic assumption behind and motivation for using external resources is that there are instances in the data where contextual clues do not provide sufficient evidence for confident classification. In such cases external resources may bridge the gap, either in the form of word lists known to refer to genes (gazetteers) or through examination of other contexts in which the same token appears and the exploitation of other more indicative contexts (as with web-querying and use of surrounding text such as abstracts).

All external resources are vulnerable to incompleteness, noise, and ambiguity. Gazetteers are arguably subject to all three and yet have been used successfully in a number of systems. Because of its size (on 26.02.2004, Google estimated that it indexed over 4,285M web pages), the web is the least vulnerable to incompleteness but is highly vulnerable to noise. Nevertheless, the web has been used to good effect in various NLP tasks (see [11] for an overview) from machine translation [12] to anaphora resolution [13]. Abstracts do not contain indicative contexts as frequently because they are so short; however their information is least vulnerable to ambiguity because a token used repeatedly within a text is likely used with the same meaning each time. Information on a word's classification elsewhere in the same text has been successfully used in a number of NER systems (cf. [14] and [15]). By incorporating all of these resources as features in a probabilistic system, we aimed to make use of their information while taking into account their reliability.

Our gazetteer was compiled from lists of gene names from biomedical sites on the Web (such as Locus Link) as well as from the Gene Ontology and the data provided for Tasks 1A and 1B. The gazetteer was cleaned by removing single character entries ("A", "1"), entries containing only digits or symbols and digits ("37", "3-1"), and entries containing only words that could be found in the English dictionary CELEX ("abnormal", "brain tumour"). The final gazetteer contained 1,731,581 entries. As stated above, gazetteer lookup was performed for each token in the preprocessing stage. Lookup was case-insensitive but punctuation was required to match exactly. For multiple word entries in the gazetteer we required all words in the entry to match. We also experimented with fuzzy-matching where each gazetteer entry was converted into a regular expression; however this matching led to inferior results and was therefore not used.

For using the web we built several contexts indicative of gene entities including "X gene", "X mutation" or "X antagonist". For each entity X identified as a gene by an initial run of the tagger, we submitted the instantiation of each pattern to the Web using the Google API and obtained the number of hits. If at least one of the patterns returned more than zero hits, the string was assigned a 'web' value for the Web feature. The classifier was then run again; this time incorporating the web feature. Using web-querying only on likely candidates for genes as identified by an initial run of the tagger was more efficient than using it on all words. Note however that this approach uses the web only to eliminate false positives and therefore does not improve recall. In other work [16] we have explored using the web with low-frequency words to improve both recall and precision.

To give a bigger context, we automatically located the full Medline abstract from which each BioCreative sentence was taken by searching Medline for the sentence using cgi scripts. (In a practical application this would be unnecessary since one would almost always have the full abstract and not a single sentence.) We incorporated additional information by tagging the abstract and then adding to words in the test sentence a feature that indicated whether the word was tagged as a gene in the abstract. We found that this feature was only helpful when combined with other information such as frequency and whether the word had appeared in the English dictionary CELEX. Presumably this was due to common words for which the abstract feature was misleading; the fact that the word "gene" was tagged as a gene in the phrase "CPA1 gene" does not indicate that it is a gene named entity in the phrase "a gene".

The final two external resources that we incorporated were the ABGene tagger [17] and the GENIA corpus [9]. We found that while the ABGene tagger used alone achieved only a modest f-score of 0.62 on the BioCreative development data, use of ABGene NE output as a feature nevertheless slightly improved our recall and overall f-score. We assume that this is because its use allowed our classifier to partially exploit the various gazetteers and lists of good and bad terms incorporated into the ABGene system (see [17]), thereby gaining additional knowledge of gene names independent of context. We also sought to incorporate the GENIA corpus of NE-annotated MEDLINE abstracts but found this difficult because it used an entirely different tag set to the BioCreative data and the mapping between them was unclear. We gained a small improvement by training the C&C tagger [15] on the full NE tag set of the GENIA corpus (consisting of 37 biomedical NEs including "cell type" and "protein molecule"), then using this tagger to tag both training and test data and using its output as a feature in our final tagger. The C&C tagger is another maximum entropy sequence tagger; it was used here for pragmatic reasons related to memory use.

### Training

As previously stated, maximum entropy systems allow incorporation of large numbers of diverse features; however, parameter estimation for large models can be time-consuming. We found that a particularly large number of features was necessary for high performance in the biomedical domain, and improved on our initial parameter estimation method (conjugate gradient descent as in [2]) by implementing a quasi-Newton optimization procedure. Quasi-Newton or limited memory variable metric methods have been shown to be faster than other algorithms by a factor of up to 7 to 1 [18]. Our final system was trained on the combined training and development data of 10,000 sentences and 262,139 words and employed approximately 1.25 million features; using quasi-Newton it trained in less than two hours. In a real-world application the time taken for training is largely irrelevant because it is a one-time cost. However, in tuning a system, training must be fast enough to allow experimentation with various configurations.

### Tagging

Tagging used a Viterbi-style algorithm with a beam size of 30. Tagging was quick; the evaluation data of 5000 sentences was tagged in approximately one minute (excluding web statistics, which were pre-computed).

### Postprocessing

We found that many of our errors stemmed from gene boundaries (37% of false positives and 39% of false negatives) and addressed this issue in several ways. Boundary errors were often due to mismatched parentheses; the parentheses-matching feature described above did not eliminate these errors due to (generally erroneous) instances in the training data which contained mismatched parentheses. We therefore used the Unix command grep to remove genes containing mismatched parentheses from our results. We also found that we obtained different gene boundaries when we ran the classifier forwards versus backwards (reversing the order of the words) and obtained a significant improvement in recall at the expense of precision by simply combining the two sets of results. This new, larger set of genes contained instances where one gene was a substring of another gene. In those instances we kept only the shorter gene. We found that this postprocessing was quite valuable and added approximately 1% to our f-score. It was used in both the open and closed sections. This postprocessing is effectively a very simple form of classifier combination, and we believe that most of the benefit comes from the classifier combination, rather than mitigating "label bias" problems [19], which tend to become very weak when rich contextual features are employed. See [20] for a more general classifier combination approach that includes forwards and backwards component models.

## Results

Tables 2,3,4 show the performance of both the "open" and "closed" versions of the system on the development and evaluation data as well as lesion studies showing the individual contribution of feature classes to the overall performance. Surprisingly, the "closed" version of the system achieves performance only 1% lower than the "open" version on the evaluation data (2% on the development data). We had expected more value from extra data sources, but it may well be that they are difficult to exploit effectively because of subtly different decisions about what does and does not count as a named entity to be tagged. However, it is also worth noting that a 1–2% improvement is relatively significant; as the performance of the classifier gradually improved during development, the improvements from revisions became progressively smaller so that at times features were incorporated which added only a tenth of a point. Also surprising was that removing word shape features actually increased our f-score by 0.13%. The "zero-order" and "first-order" experiments refer to how far back the classifier can see the NE tags assigned to previous words during sequence search. Thus a zero-order model can only see the classification of the current word, while a first-order model can see the classification assigned to the previous word (but not the words before). Removing second and third order features also improved our result marginally.

## Discussion

### Sources of error

A number of false positives (FPs) occurred when the entity tagged by the classifier was a description of a gene rather than a gene name, as with "homologue gene". FPs also occurred with several strings that were composed of characters and digits or sequences of capitalised letters, or that included symbols and punctuation. This occurred frequently with measures, such as "kat/L" (katal per litre) and acronyms for non-gene entities. Acronym ambiguity was a related source of error. The abbreviation "HAT", for instance, could stand for the gene name "histone acetyltransferase" but actually referred to "hepatic artery thrombosis" in one specific context.

False negatives (FNs) were frequently caused by gene names that had not been encountered in the training data, so that the classifier did not have information about them and contextual clues were insufficient. FNs also occurred in some coordinated NPs where the modifier was attached to only one of the phrases but modified all of the coordinated members. Abbreviations, expansions, and names in parentheses were also frequent causes of FNs.

The single largest source of error was mistaken boundaries (37% of FP and 39% of FN). In most cases, the classifier identified one correct and one incorrect boundary (i.e. either the beginning or the end). It often included left or right context as part of the entity which was not contained in the gold standard. In several instances, the classifier split a string into separate entities which in fact referred to a single entity, or tagged separate entities as a single one. Tokenisation errors sometimes triggered boundary errors, as with "PGS-2 . CAT reporter gene" where the classifier only recognized "CAT reporter" as a gene. Because many abbreviations were not genes and because the precision and recall of the gazetteer were fairly low, we believe that both abbreviation and gazetteer features helped more in identifying gene boundaries than in identifying genes.

Some of our errors were due to errors in the evaluation data. In example (1) below which appeared in the evaluation data, our system annotated "nuclear factor Y" as a gene while the gold standard annotated only "nuclear factor"; we were penalized for both a FP and a FN. This appears to be an error and is inconsistent with (2) which appeared in the training data. Examples (3) and (4) also appear to be misannotated; a quick web search shows that SGOT (our system's FP) in (3) is a well-known enzyme, while the GaAs/(Al,Ga)As heterojunctions (our system's FN) in (4) are found in semiconductors. Even in cases where our error in the evaluation data was in fact an error, it could not infrequently be traced to a similar error in the training data. In example (5) we annotated "human cyclin-dependent kinase" and were penalized for a FP; however, our annotation mirrors the pattern of (6) which appeared in the training data.

(1) ...both PC12 and C6 cell nuclear extracts were recruited by the CCAAT-box as a complex containing *nuclear factor *Y.

(2) The sequence-specific interaction of *nuclear factor HiNF-D *with this key proximal promoter element of the *H4-FO108 gene *is cell cycle regulated in normal diploid cells

(3) Nitrogen balance was compared, and metabolic complications were monitored by evaluating BUN, serum creatinine, creatinine clearance, serum CO2, SGOT, SGPT, *serum LDH*, and *serum alkaline phosphatase*.

(4) Envelope-function matching conditions for *GaAs/(Al,Ga)As heterojunctions*.

(5) Structure of the gene encoding the *human cyclin-dependent kinase inhibitor p18*

(6) ...which targets the *cyclin-dependent kinase *(*Cdk*) inhibitor *Sic1p*...

### Directions for improvement

**Figure 1:**
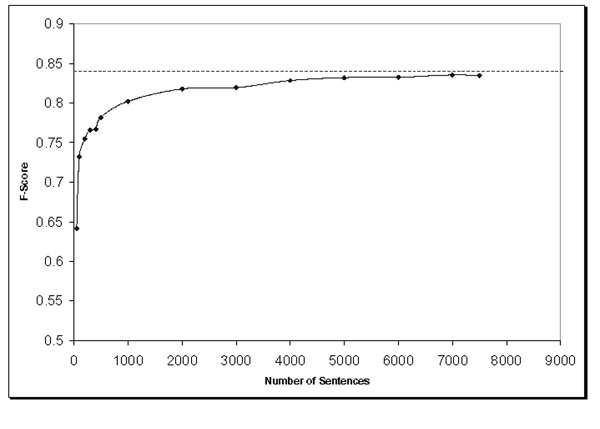
**Learning curve. **Learning curve for the performance of the "open" NER system on development data.

The learning curve in Figure 1 suggests that we can expect only very limited improvement from the availability of additional training data, given the current task and feature set. Rather we must explore other avenues, including better exploitation of existing features and resources, development of additional features, incorporation of additional external resources, or experimentation with other algorithms and strategies for approaching the task.

One obvious improvement of our current system would be the incorporation of protein names into our gazetteer. Due to ambiguity in the guidelines we were unaware that protein names were to be recognized and incorporated only gene names into our gazetteer.

Secondly, more careful attention to coordination may improve results. This could involve parsing or less sophisticated treatment of coordinations. Our work in [16] shows that full parsing can give value to NER tasks. However, if one heads in this direction, one can no longer so easily think of NER as a lightweight initial processing step feeding into more complex analysis such as information extraction and full sentence understanding.

Thirdly, boundary errors might be addressed more effectively with a more sophisticated post-processing stage. Considering only the problem of segmentation of NEs, Collins [21] applies reranking to candidate structures generated from a maximum-entropy tagger and achieves a 17.7% relative reduction in error rate. He used reranking to allow features that describe the full NE identified by the tagger, such as its first and last words and attributes thereof, and whether all words between a set of quotes were given the same tag (reminiscent of the parentheses problems in our data). Such features cannot be encoded in a standard sequence tagger.

Another possible avenue would be automatic addition of conjunctions of current features [22,23]. A number of the features listed in Table 1, as well as the features used to incorporate external resources, are relatively unintuitive conjunctions of other features that were chosen by lengthy trial and error processes. Feature induction might suggest useful feature conjunctions that we have overlooked and reduce the cost of incorporating additional resources. All told, we spent about 25 person-weeks extending the system of [2] for this evaluation exercise, much of it in designing and testing variant feature sets. This leaves us open to the criticism that much of the effort was not machine learning, and one might have been able to develop a system of hand-crafted rules in the same time. Use of automatic feature induction would partly address this criticism.

Finally, improvements in the annotation of data used for both training and evaluation may be the single best source of improvement. We note that the quality of data for BioCreative was overall quite good and the organizers' innovation of providing alternate correct boundaries for a given named entity was instrumental in reducing spurious errors due to debatable boundaries. However, as noted in the previous section, a significant proportion of errors could be attributed to errors in the annotated data, and the fact that no clear annotation guidelines were provided in a domain as complex as molecular biology would suggest that there is room for improvement.

## Conclusion

We have presented in detail a machine learning system for identifying genes and proteins in text and described its feature set comprising both contextual clues and external resources. We have also presented its performance on the BioCreative development and evaluation data, analyzed its sources of error, and identified possible avenues for improvement.

Many of our features were focused on increasing the correct identification of entity boundaries. This is partly an artifact of the scoring metric: using an f-score of exact match precision and recall means that one is penalized twice, both for a FP and a FN, in cases of an incorrect boundary identification. One scores better in such cases if one suggests no entity. This problem was somewhat ameliorated within the BioCreative evaluation by a facility for annotators to be able to specify alternate correct answers, which allowed as correct matches of several lengths in places where the annotators thought it appropriate. The CoNLL task also used a straight f-score metric, but note that the "mid-nineties" results commonly remembered from MUC NER competitions reflect an easier metric where partial credit was given for cases of incorrect boundary identification. We evaluated our BioCreative result of 83.2 with the MUC scorer and scored 85.62. Nevertheless, the lower results equally reflect that finding correct entity boundaries in the biomedical domain is an extremely hard task, whereas in many cases it is quite trivial for people or place names in English – capitalization giving sufficient clues.

The final performance of the tagger at 0.83 f-score remains far below the best results reported for the most well-researched NER task of PERSON, LOCATION, and ORGANIZATION entities in newswire texts. Using the set of features designed for that task in CoNLL 2003 [24], our system achieves an f-score of 0.76 on the BioCreative development data, a dramatic ten points lower than its f-score of 0.86 on the CoNLL newswire data. Despite the massive size of the final feature set (almost twice as many features as used for CoNLL), its final performance of 0.83 is still below its performance on the CoNLL data (and far below the 0.89 f-score of the top-performing system in the CoNLL task), even though the BIOCREATIVE task involved only one distinction. The discrepancy in performance is a striking illustration of the greater difficulty of NER in the biomedical domain.

It is worth comparing these performance figures with levels of interannotator agreement in the biomedical domain. Interannotator agreement effectively provides a ceiling on the performance that can be expected from a system by measuring how well a human annotator performs on a task. While agreement for the MUC entities was measured at 97% (though again using the slightly more lenient measure implemented in the MUC scorer), a number of results have measured agreement for biomedical NEs to be substantially lower, with f-scores in the range of 0.87 [25] to 0.89 [26]. With interannotator agreement so low, it appears that we cannot currently expect to improve system performance more than a few points. This suggests that more clarity in what should be annotated (or perhaps just when a variety of answers of different extent should be counted as correct) is needed. It also may suggest that performance of 83% or improvement of just a few points is sufficient for the technology to be practically applicable.

## Figures and Tables

**Table 1:** Features Used Description of the Full Feature Set Used In the Closed Section Submission.

Word Features	w_*i*_
	w_*i*-1_
	w_*i*+1_
	Last "real" word
	Next "real" word
	Disjunction of 4 previous words
	Disjunction of 4 next words
Bigrams	w_*i *_+ w_*i*-1_
	w_*i *_+ w_*i*+1_

TnT POS	POS_*i*_
	POS_*i*-1_
	POS_*i*+1_

Character Substrings	Up to a length of 6

Abbreviations	abbr_*i*_
	abbr_*i*-1 _+ abbr_*i*_
	abbr_*i *_+ abbr_*i*+1_
	abbr_*i*-1 _+ abbr_*i *_+ abbr_*i*+1_

Word Shape	shape_*i*_
	shape_*i*-1_
	shape_*i*+1_
	shape_*i*-1 _+ shape_*i*_
	shape_*i *_+ shape_*i*+1_
	shape_*i*-1 _+ shape_*i *_+ shape_*i*+1_

Previous NE	NE_*i*-1_
	NE_*i*-2 _+ NE_*i*-1_

Previous NE + Word	NE_*i*-1 _+ w_*i*_

Previous NE + POS	NE_*i*-1 _+ POS_*i*-1 _+ POS_*i*_
	NE_*i*-2 _+ NE_*i*-1 _+ POS_*i*-2 _+ POS_*i*-1 _+ POS_*i*_

Previous NE + Shape	NE_*i*-1 _+ shape_*i*_
	NE_*i*-1 _+ shape_*i*+1_
	NE_*i*-1 _+ shape_*i*-1 _+ shape_*i*_
	NE_*i*-2 _+ NE_*i*-1 _+ shape_*i*-2 _+ shape_*i*-1 _+ shape_*i*_

Paren-Matching	A feature that signals when one parentheses in a pair has been assigned a different tag than the other in a window of 4 words

**Table 2:** Development set results System Results on Cross-Validated Training/Dev Data.

	Precision	Recall	F-Score
Open	0.813	0.861	0.836
Closed	0.784	0.852	0.817

**Table 3:** Test set results System Results on Evaluation Data.

	Precision	Recall	F-Score
Open	0.828	0.835	0.832
Closed	0.792	0.854	0.822

**Table 4:** Lesion study results Results on Cross-Validated Training and Development Data With One Feature Removed At a Time

	Precision	Recall	F-Score	Δ F
Abbreviations	0.813	0.860	0.836	-0.05%
Abgene	0.810	0.861	0.834	-0.18%
Abstract	0.811	0.855	0.832	-0.39%
Gazette	0.807	0.857	0.831	-0.51%
Genia	0.806	0.857	0.831	-0.55%
Substrings	0.814	0.852	0.833	-0.37%
POS_*i*,*i*-1,*i*+1_	0.814	0.860	0.836	-0.03%
Google Web	0.807	0.864	0.835	-0.17%
Word Shape	0.815	0.862	0.838	+0.13%
Zero Order	0.741	0.799	0.770	-6.66%
First Order	0.818	0.853	0.835	-0.15%
Second Order	0.814	0.861	0.837	+0.06%
Third Order	0.814	0.863	0.837	+0.07%

**Table 5:** Examples of Errors Examples of FPs, FNs and boundary errors. In some of the examples square brackets are used to indicate the differences between the classifier's output and the annotation in the gold standard.

**False Positives**	**Classifier (CL)**	**Gold Standard (GS)**
General Words	homolog gene	-
Measures	kat/L	-
Possible Errors in GS	[ssDNA-] and [RNA-binding protein]	ssDNA- and [RNA-binding protein]

**False Negatives**	**Classifier (CL)**	**Gold Standard (GS)**

Coordination	[YAP2 uORF1] and uORF2	[YAP2 uORF1] and [uORF2]
Missing Expansion	zinc-finger protein ([THZif-1])	[zinc-finger protein] ([THZif-1])

**Boundary Errors**	**Classifier (CL)**	**Gold Standard (GS)**

GS NE contains CL NE(s)	AP-1 complexes	high mobility AP-1 complexes
	USH1C	USH1C disease gene
	partner of [Rac]	[partner of Rac]

CL NE contains GS NE(s)	regulator virF	virF
	Wnt pathway	Wnt
CL and GS Overlap	Serum [Fibrin Degradation Products]	[Serum Fibrin] Degradation Products
